# Crystal structure and Hirshfeld surface analysis of 3-(2-chloro-6-fluoro­phen­yl)-1,5-bis­(thio­phen-2-yl)pentane-1,5-dione

**DOI:** 10.1107/S2056989025009284

**Published:** 2025-10-31

**Authors:** Atash V. Gurbanov, Mehmet Akkurt, Nurlana D. Sadikhova, Gizachew Mulugeta Manahelohe

**Affiliations:** a Excellence Center, Baku State University, Z. Khalilov Str. 33, AZ 1148, Baku, Azerbaijan; b Department of Physics, Faculty of Sciences, Erciyes University, 38039 Kayseri, Türkiye; c Department of Organic Chemistry, Baku State University, Z. Khalilov Str. 33, AZ 1148, Baku, Azerbaijan; d Department of Chemistry, University of Gondar, PO Box 196, Gondar, Ethiopia; Harvard University, USA

**Keywords:** crystal structure, 2-chloro-6-fluoro­phenyl ring, thio­phene rings, disorder, Hirshfeld surface analysis

## Abstract

In the title compound, C_19_H_14_ClFO_2_S_2_, the mol­ecular conformation is stabilized by intra­molecular C—H⋯F, C—H⋯Cl and C—H⋯O hydrogen bonds. In the crystal, the mol­ecules are linked into [010] chains by C—H⋯O hydrogen bonds, creating a *C*(6) motif and weak C—H⋯F and C—H⋯Cl inter­actions link these chains into sheets parallel to the (100) plane. The entire –C_6_H_3_FCl group is disordered over two positions in a 0.931 (4):0.069 (4) ratio. A Hirshfeld surface analysis indicates that the most important contributions to the crystal packing are from H⋯H (26.7%), C⋯H/H⋯C (17.2%), S⋯H/H⋯S (15.0%) and O⋯H/H⋯O (12.0%) inter­actions.

## Abstract 2

In the crystal, the mol­ecules are linked into [010] chains by C—H⋯O hydrogen bonds, creating a *C*(6) motif. Weak C—H⋯Cl and C—H⋯F inter­actions link these chains, forming sheets parallel to the (100) plane.

## 1. Chemical context

Substituted thio­phenes are important inter­mediates in organic synthesis, and are widely used in medicine, industrial chemistry, and materials science (Peng *et al.*, 2024 ▸). Thio­phenes with anti-inflammatory, anti­bacterial, anti-cancer and other biological activities are widely used in the pharmaceutical industry, and a number of thio­phene-containing drugs have been approved by the Food and Drug Administration (FDA), such as Cefoxitin, Raloxifene and Suprofen (Schweizer *et al.*, 2011 ▸). In addition, thio­phenes exhibit adequate electrical conductivity because of the presence of sulfur atoms. In particular, polythio­phenes have found applications in organic light-emitting diodes, organic semiconductors, field effect transistors, *etc*. (Turkoglu *et al.*, 2019 ▸; Zhang *et al.*, 2015 ▸). Functional properties of thio­phenes are strongly dependent on the inter­molecular inter­actions, such as hydrogen and chalcogen bonds (Gurbanov *et al.*, 2020 ▸, 2022 ▸; Mahmudov *et al.*, 2021 ▸, 2023 ▸). The cooperation of –Cl, –F and C=O groups with the thio­phen-2-yl synthon in thio­phenes can improve the functional properties of the corresponding organic materials (Gurbanov *et al.*, 2023 ▸; Mahmoudi *et al.*, 2017 ▸, 2018 ▸; Velásquez *et al.*, 2019 ▸). Thus, in the current work we have synthesized a new thio­phene derivative, 3-(2-chloro-6-fluoro­phen­yl)-1,5-di(thio­phen-2-yl)pentane-1,5-dione, which provides multiple inter­molecular inter­actions.

## 2. Structural commentary

**Figure 1:**
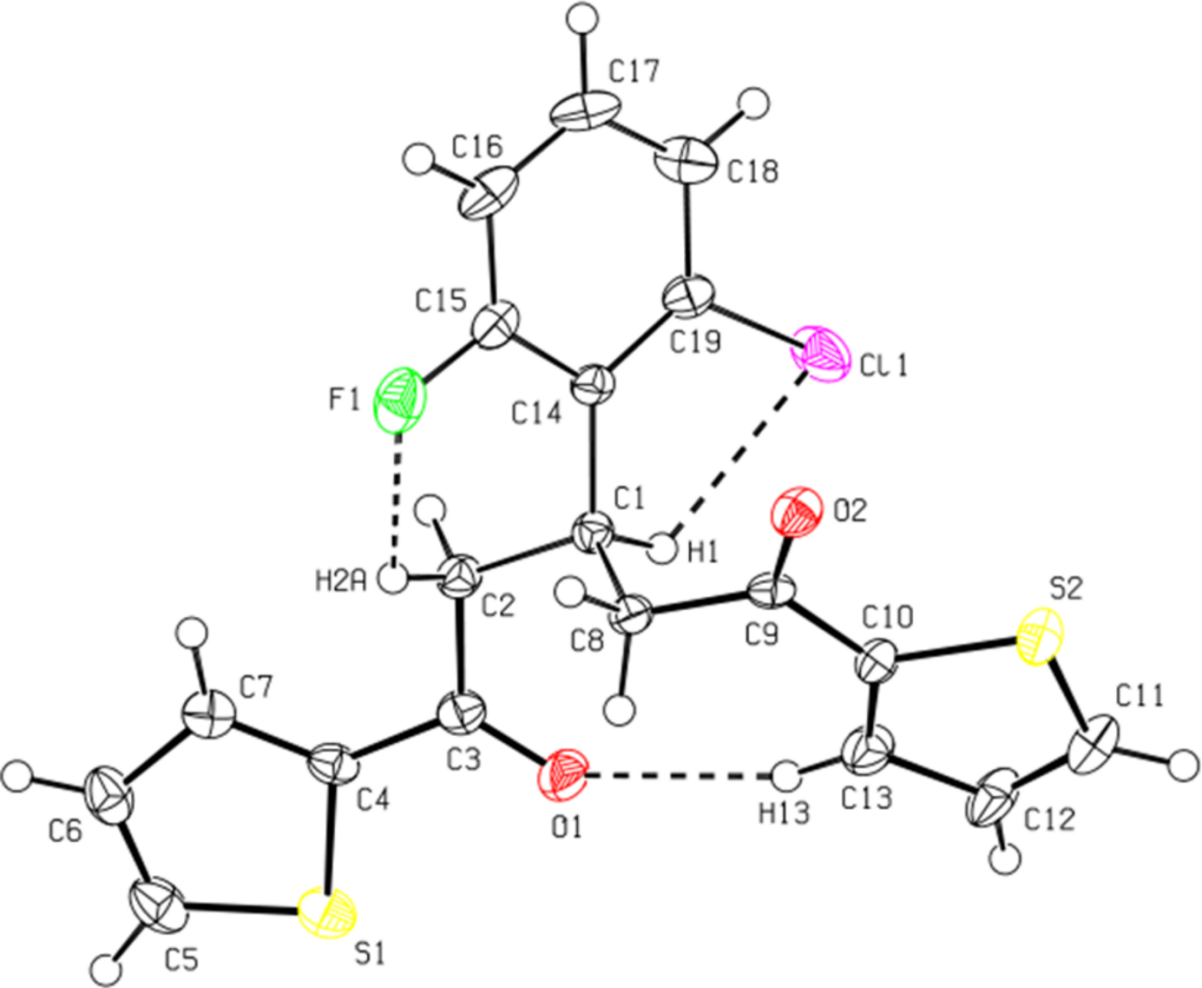
The title mol­ecule with the atom-labelling scheme and displacement ellipsoids drawn at the 50% probability level. Only the major disorder component is displayed. Intra­molecular C—H⋯Cl, C—H⋯F, and C—H⋯O hydrogen bonds are shown as dashed lines.

Intra­molecular C2—H2*A*⋯F1, C1—H1⋯Cl1 and C13—H13⋯O1 inter­actions (Fig. 1 ▸, Table 1 ▸) maintain the mol­ecular conformation of the major disorder component of the title compound, resulting in *C*(6), *C*(5) and *C*(9) motifs (Bernstein *et al.*, 1995 ▸), respectively. The major and minor occupancy benzene rings (C14–C19 and C14*A*–C19*A*) of the disordered 2-chloro-6-fluoro­phenyl group form an angle of 3.3 (5)° with one another. The angles between the planes of the two thio­phen-2-yl rings (S1/C4–C7 and S2/C10–C13) and the major occupancy ring (C14–C19) of the disordered 2-chloro-6-fluoro­phenyl group are 40.9 (1) and 51.6 (1)°, respectively. The dihedral angle between the two thio­phen-2-yl rings is 19.3 (1)°. The torsion angles C3—C2—C1—C14, C3—C2—C1—C8, C2—C1—C8—C9, C14—C1—C8—C9, C1—C8—C9—C10 and C8—C9—C10—C13 are 165.2 (2), −70.4 (2), 169.8 (2), −65.8 (3), −81.2 (2), and 0.4 (4)°, respectively. The sum of the angles about C1 is 333.6 (2)° for the major disorder component. All geometric parameters are normal and consistent with those of related compounds listed in the section *Database survey*.
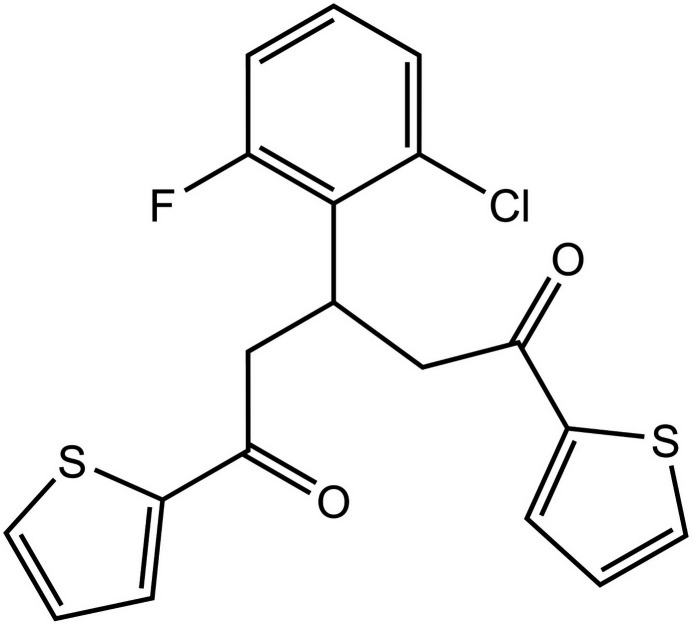


## 3. Supra­molecular features and Hirshfeld surface analysis

**Figure 2:**
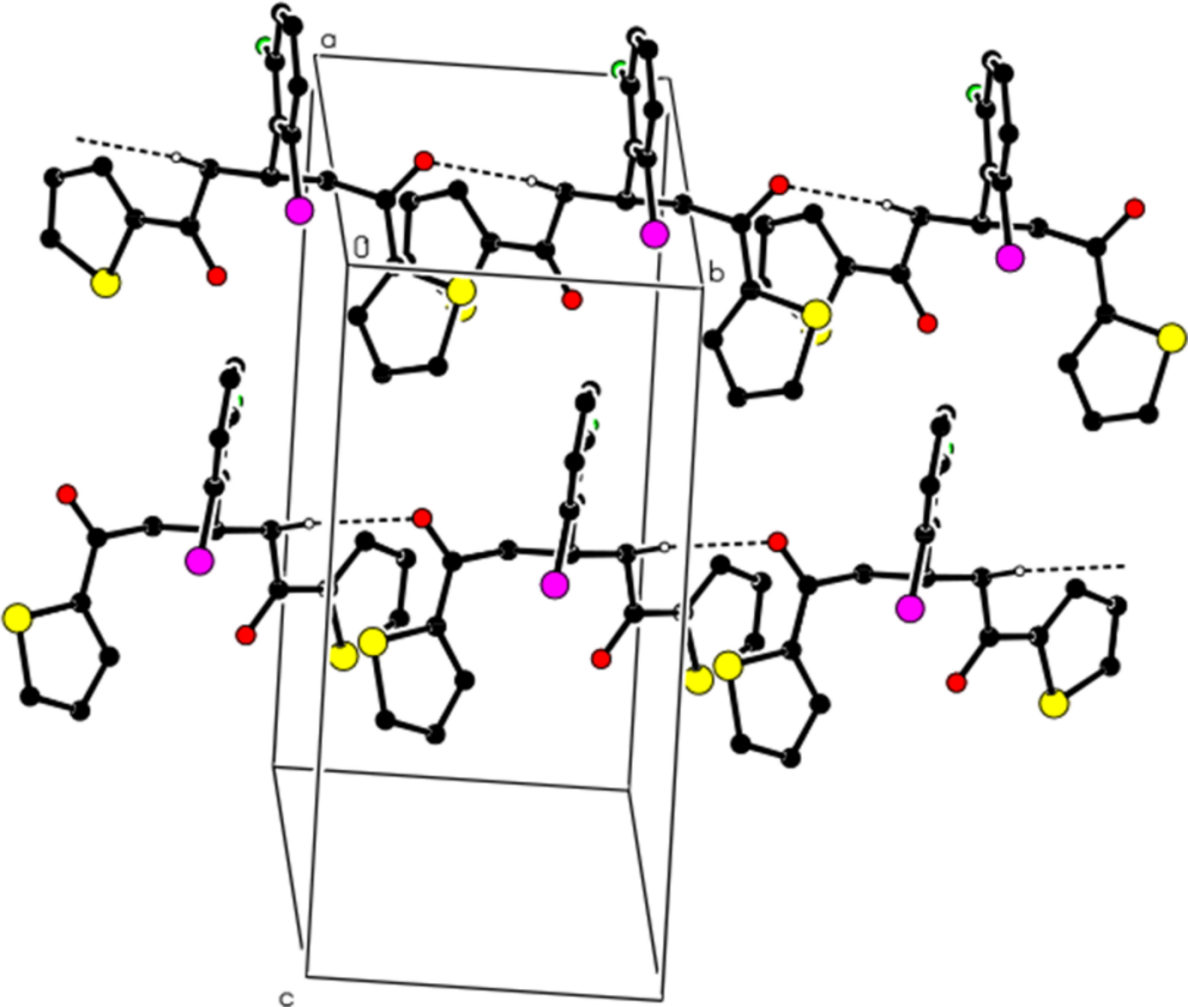
A partial packing diagram showing the unit cell. Dashed lines indicate C—H⋯O hydrogen bonds. Only H atoms involved in the hydrogen bonds and the major disorder component are shown for clarity.

In the crystal, C—H⋯O hydrogen bonds (Table 1 ▸) link the mol­ecules into chains extending along the [010] direction (Fig. 2 ▸) and weak C—H⋯F and C—H⋯Cl inter­actions (Table 1 ▸) further link these chains into sheets parallel to the (100) plane (Figs. 1 ▸ and 2 ▸ in the supporting information). van der Waals inter­actions between the sheets also contribute to the cohesion of the mol­ecular packing. No π–π or C—H⋯π inter­actions are found.

**Figure 3:**
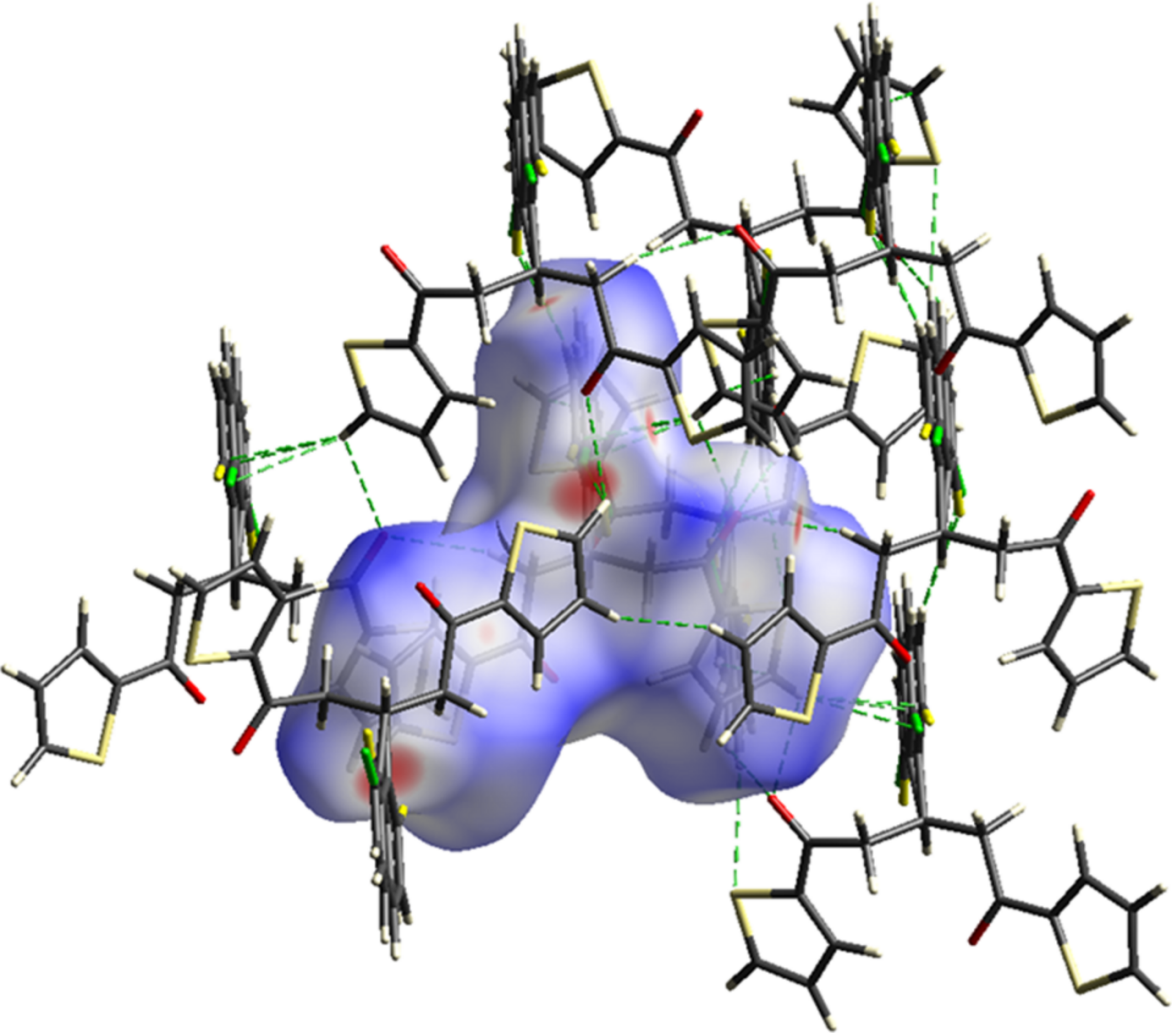
View of the three-dimensional Hirshfeld surface of the compound plotted over *d*_norm_.

**Figure 4:**
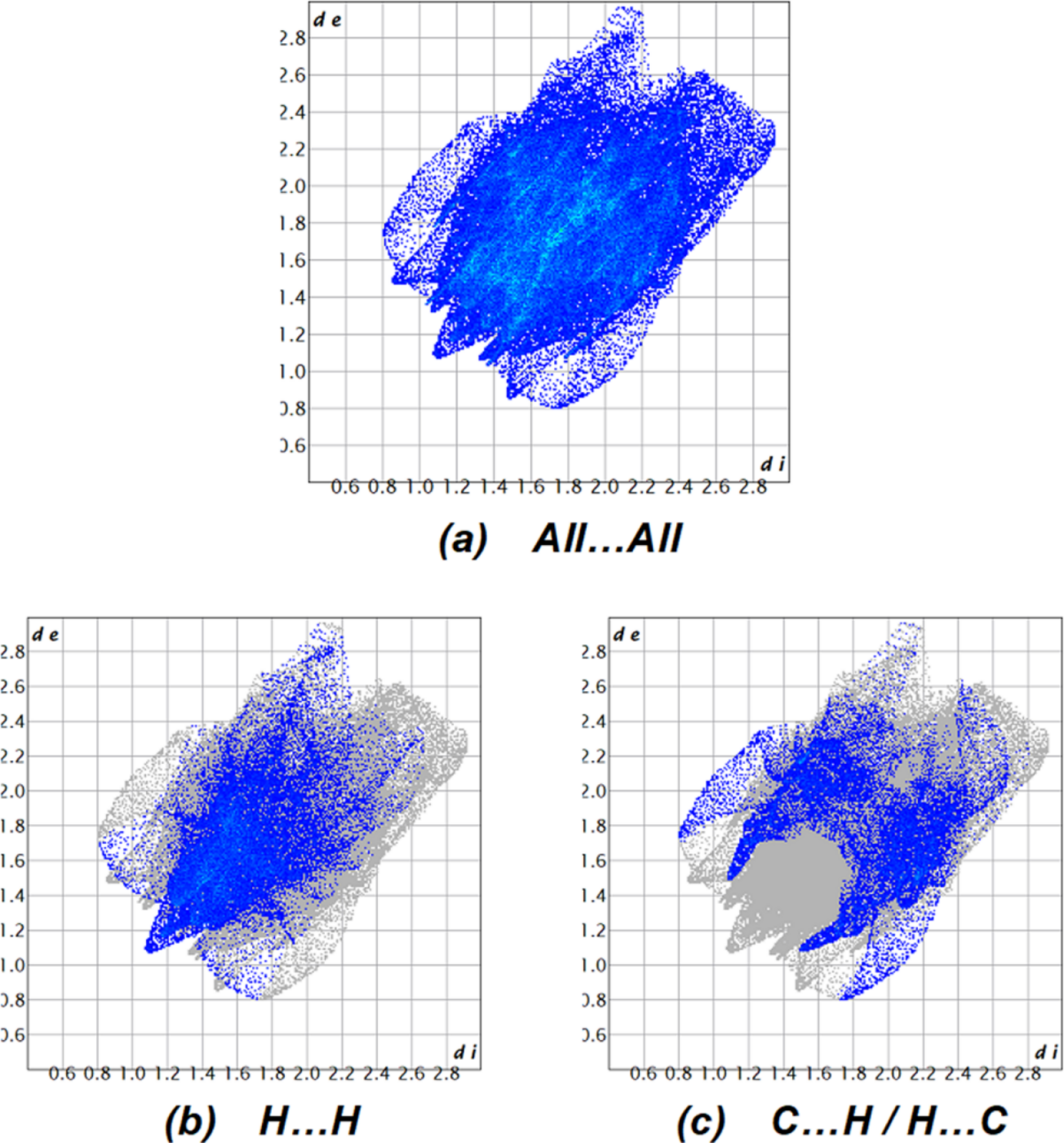
The two-dimensional fingerprint plots, showing (*a*) all inter­actions, and delineated into (*b*) H⋯H and (*c*) C⋯H/H⋯C inter­actions [*d*_e_ and *d*_i_ represent the distances from a point on the Hirshfeld surface to the nearest atoms outside (external) and inside (inter­nal) the surface, respectively].

We carried out a Hirshfeld surface analysis to further investigate the inter­molecular inter­actions using *Crystal Explorer 17.5* (Spackman *et al.*, 2021 ▸). The Hirshfeld surface mapped over *d*_norm_ is illustrated in Fig. 3 ▸. The red spots on the Hirshfeld surface plot indicate the inter­molecular C2—H2*B*⋯O2, C5—H5⋯Cl1*A* and C16—H16⋯F1A contacts shown in Table 1 ▸. The overall two-dimensional fingerprint plot is shown in Fig. 4 ▸*a*. The Hirshfeld surface analysis reveals that H⋯H (26.7%) and C⋯H/H⋯C (17.2%) contacts are the main contributors to the crystal packing (Tables 1 ▸ and 2 ▸; Fig. 4 ▸*b*–*c*), followed by S⋯H/H⋯S (15.0%), O⋯H/H⋯O (12.0%), F⋯H/H⋯F (7.6%) and Cl⋯H/H⋯Cl (6.4%) contacts. The other minor contributions are less than 4.5%. The Hirshfeld surface analysis confirms the importance of H-atom contacts in the crystal (Hathwar *et al.*, 2015 ▸).

## 4. Database survey

A search of the Cambridge Structural Database (CSD, Version 6.00, update of April 2025; Groom *et al.*, 2016 ▸) found that the most closely related structures containing the 4-(2-chloro-6-fluoro­phen­yl)-1,7-di(thio­phen-2-yl)heptane-1,7-di­one unit are MIGVEE (Butcher *et al.*, 2007 ▸), WEPJOR (Yathirajan *et al.*, 2006 ▸) and XUJRAZ (Sharmoukh *et al.*, 2025 ▸).

The angles between the plane of the benzene ring and those of the two thio­phene rings are 89.7 (5) and 63.7 (1)° for MIGVEE, 84.9 (2) and 68.8 (2) ° for WEPJOR, and 75.27 (5) and 83.8 (2)° for XUJRAZ. In MIGVEE, the mol­ecules are linked by C—Br⋯π and C—Cl⋯π inter­actions and there are no classical hydrogen bonds. In WEPJOR, the mol­ecules form layers parallel to (200) through C—H⋯O hydrogen bonds. C—H⋯π, C—Br⋯π, and C—O⋯π inter­actions formed between the layers strengthen the mol­ecular packing. In the crystal of XUJRAZ, C—H⋯O hydrogen bonds form chains of mol­ecules extending along the *c*-axis direction. These are linked by C—H⋯S hydrogen bonds and C—H⋯π inter­actions into corrugated layers parallel to the *bc* plane.

## 5. Synthesis and crystallization

**Figure 5:**
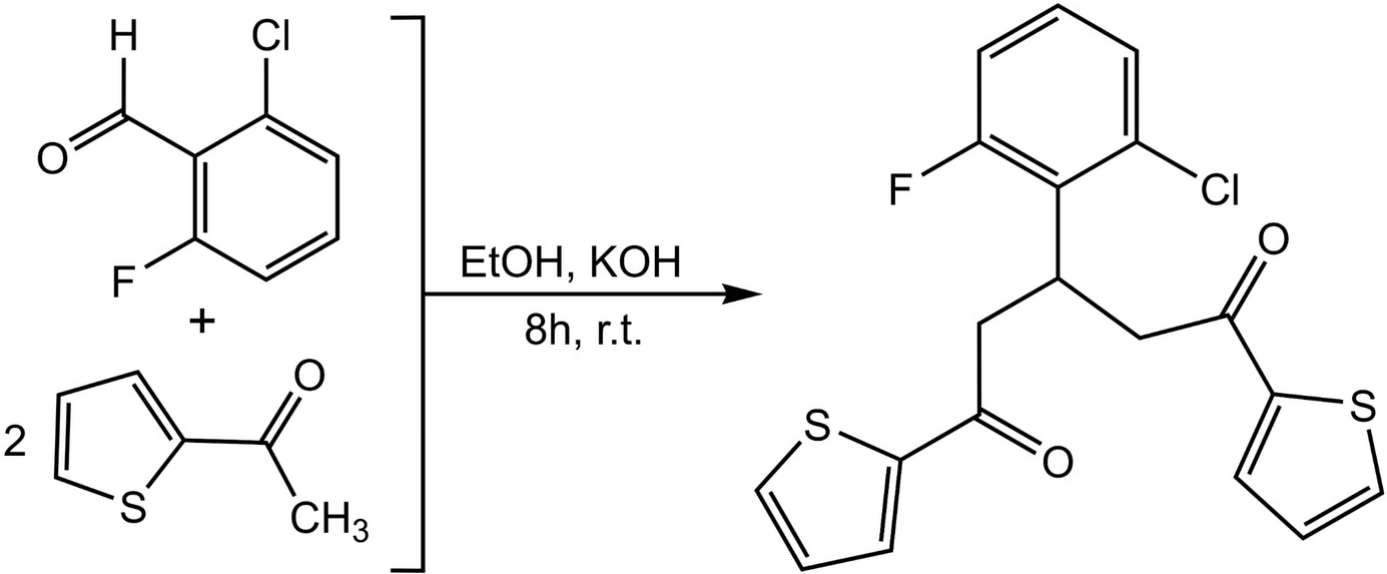
Synthesis of 3-(2-chloro-6-fluoro­phen­yl)-1,5-di(thio­phen-2-yl)pentane-1,5-dione.

2-Chloro-6-fluoro­benzaldehyde (10 mmol) was added into a solution of 1-(thio­phen-2-yl)ethan-1-one (20 mmol) in EtOH (60 mL). KOH pellets (25 mmol) were then added to the solution. The solution was stirred at room temperature for 8 h. The off-colourless solid was collected by filtration and washed with EtOH (3 × 10 mL). Recrystallization from CHCl_3_–MeOH afforded a white crystalline solid of the title compound (yield, 52%). The synthesis is shown in Fig. 5 ▸. Analysis calculated for C_19_H_14_ClFO_2_S_2_: C, 58.09; H, 3.59. Found: C, 58.05; H, 3.55. ^1^H NMR (500 MHz, CDCl_3_): δ (ppm) 7.83 (*dd*, *J* = 1.0, 3.8 Hz, 2H, ArH), 7.68 (*dd*, *J* = 1.0, 4.9 Hz, 2H, ArH), 7.55–7.52 (*m*, 2H, ArH), 7.24 (*d*, *J* = 8.0 Hz, 1H, ArH), 7.08 (*dd*, *J* = 3.9, 4.8 Hz, 2H, ArH), 4.60 (*p*, *J* = 7.2 Hz, 1H, CH), 3.51 (*dd*, *J* = 7.1, 16.9 Hz, 2H, CH_2_), 3.39 (*dd*, *J* = 7.2, 16.9 Hz, 2H, CH_2_); ^13^C{H} NMR (125 MHz, CDCl_3_): δ (ppm) 198.6, 160.0, 144.1, 136.6, 134.6, 134.4, 133.4, 128.7, 128.4, 124.2, 113.3, 45.0, 36.5.

## 6. Refinement

Crystal data, data collection and structure refinement details are summarized in Table 3 ▸. All carbon-bound H atoms were positioned geometrically and refined as riding: C—H = 0.95–1.00 Å with *U*_iso_(H) = 1.2*U*_eq_(C). The –C_6_H_3_FCl group (C14–C19/Cl1/F1) atoms and the main group atom it is attached to (C1) were treated as disordered in a ratio of 0.931 (4):0.069 (4) over two positions. Refinement was performed by substituting the majority of the fluorine and chlorine atoms in the disorder with the minor portion’s chlorine and fluorine atoms, respectively. The FLAT command was used to ensure that the atoms of the two disorder parts lie in the same plane. In the major and minor parts (C14–C19/Cl1/F1 and C14*A*–C19*A*/Cl1A/F1*A*) of the disorder group, the C—Cl and C—F bonds, as well as the corresponding C–C bond lengths (*e.g.* C14—C19 and C14A—C19*A*) of the benzene ring, and the C1—C14 and C1—C14*A* distances connecting the –C_6_H_3_FCl group to the main group, were forced to have the same value using the SADI command. Displacement parameters of similar corresponding atoms were forced to be the same using the EADP command. For the disordered main group atom (C1/C1*A*), the EXYZ and EADP commands were applied. One reflection (1 0 0), affected by the incident beam-stop was omitted in the final cycles of refinement.

## Supplementary Material

Crystal structure: contains datablock(s) I. DOI: 10.1107/S2056989025009284/oi2026sup1.cif

Structure factors: contains datablock(s) I. DOI: 10.1107/S2056989025009284/oi2026Isup2.hkl

Views of molecular packing along the [010] and [001] directions. DOI: 10.1107/S2056989025009284/oi2026sup3.pdf

Supporting information file. DOI: 10.1107/S2056989025009284/oi2026Isup4.cml

CCDC reference: 2496991

Additional supporting information:  crystallographic information; 3D view; checkCIF report

## Figures and Tables

**Table 1:** Hydrogen-bond geometry (Å, °)

*D*—H⋯*A*	*D*—H	H⋯*A*	*D*⋯*A*	*D*—H⋯*A*
C1—H1⋯Cl1	1.00	2.57	3.128 (3)	115
C1—H1⋯F1*A*	1.00	2.21	2.86 (5)	122
C2—H2*A*⋯Cl1*A*	0.99	2.31	2.91 (3)	118
C2—H2*A*⋯F1	0.99	2.32	2.898 (5)	116
C2—H2*B*⋯O2^i^	0.99	2.48	3.417 (3)	157
C5—H5⋯Cl1*A*^ii^	0.95	2.49	3.12 (3)	123
C8—H8*A*⋯Cl1*A*	0.99	2.35	3.01 (3)	123
C13—H13⋯O1	0.95	2.45	3.367 (3)	163
C16—H16⋯F1*A*^iii^	0.95	2.50	3.35 (3)	149

Symmetry codes: (i) 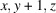; (ii) 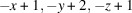; (iii) 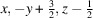.

**Table 2:** Summary of short inter­atomic contacts (Å)

Contact	Distance	Symmetry operation
H18*A*⋯H11	2.50	−*x*, 1 − *y*, 1 − *z*
F1*A*⋯H16	2.50	*x*, 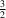 − *y*, 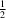 + *z*
H5⋯O1	2.64	1 − *x*, 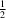 + *y*, 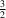 − *z*
O2⋯H2*B*	2.48	*x*, −1 + *y*, *z*
O2⋯H17*A*	2.44	−*x*, − 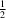 + *y*, 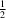 − *z*
H5⋯Cl1*A*	2.49	1 − *x*, 2 − *y*, 1 − *z*
O2⋯H11	2.68	*x*, 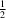 − *y*, 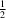 + *z*
H6⋯H6	2.32	1 − *x*, 3 − *y*, 1 − *z*

**Table 3:** Experimental details

Crystal data	
Chemical formula	C_19_H_14_ClFO_2_S_2_
*M*_r_	392.87
Crystal system, space group	Monoclinic, *P*2_1_/*c*
Temperature (K)	150
*a*, *b*, *c* (Å)	16.4479 (14), 7.6867 (6), 15.4738 (12)
β (°)	113.697 (2)
*V* (Å^3^)	1791.4 (3)
*Z*	4
Radiation type	Mo *K*α
μ (mm^−1^)	0.47
Crystal size (mm)	0.28 × 0.21 × 0.11

Data collection	
Diffractometer	Bruker APEXII CCD
Absorption correction	Multi-scan (*SADABS*; Krause *et al.*, 2015 ▸)
*T*_min_, *T*_max_	0.868, 0.936
No. of measured, independent and observed [*I* > 2σ(*I*)] reflections	24966, 3678, 2523
*R*_int_	0.069
(sin θ/λ)_max_ (Å^−1^)	0.626

Refinement	
*R*[*F*^2^ > 2σ(*F*^2^)], *wR*(*F*^2^), *S*	0.043, 0.101, 1.02
No. of reflections	3678
No. of parameters	251
No. of restraints	19
H-atom treatment	H-atom parameters constrained
Δρ_max_, Δρ_min_ (e Å^−3^)	0.36, −0.33

Computer programs: *APEX4* and *SAINT* (Bruker, 2012 ▸), *SHELXS97* (Sheldrick, 2008 ▸), *SHELXL2014* (Sheldrick, 2015 ▸), *ORTEP-3 for Windows* (Farrugia, 2012 ▸) and *PLATON* (Spek, 2020 ▸).

## supplementary crystallographic information

### 3-(2-Chloro-6-fluorophenyl)-1,5-bis(thiophen-2-yl)pentane-1,5-dione Crystal data

**Table:**

C_19_H_14_ClFO_2_S_2_	*F*(000) = 808
*M**_r_* = 392.87	*D*_x_ = 1.457 Mg m^−^^3^
Monoclinic, *P*2_1_/*c*	Mo *K*α radiation, λ = 0.71073 Å
*a* = 16.4479 (14) Å	Cell parameters from 3391 reflections
*b* = 7.6867 (6) Å	θ = 2.7–22.6°
*c* = 15.4738 (12) Å	µ = 0.47 mm^−^^1^
β = 113.697 (2)°	*T* = 150 K
*V* = 1791.4 (3) Å^3^	Plate, colourless
*Z* = 4	0.28 × 0.21 × 0.11 mm

### 3-(2-Chloro-6-fluorophenyl)-1,5-bis(thiophen-2-yl)pentane-1,5-dione Data collection

**Table:**

Bruker APEXII CCD diffractometer	2523 reflections with *I* > 2σ(*I*)
φ and ω scans	*R*_int_ = 0.069
Absorption correction: multi-scan (SADABS; Krause *et al.*, 2015)	θ_max_ = 26.4°, θ_min_ = 2.6°
*T*_min_ = 0.868, *T*_max_ = 0.936	*h* = −12→20
24966 measured reflections	*k* = −9→9
3678 independent reflections	*l* = −19→19

### 3-(2-Chloro-6-fluorophenyl)-1,5-bis(thiophen-2-yl)pentane-1,5-dione Refinement

**Table:**

Refinement on *F*^2^	Primary atom site location: structure-invariant direct methods
Least-squares matrix: full	Secondary atom site location: difference Fourier map
*R*[*F*^2^ > 2σ(*F*^2^)] = 0.043	Hydrogen site location: inferred from neighbouring sites
*wR*(*F*^2^) = 0.101	H-atom parameters constrained
*S* = 1.02	*w* = 1/[σ^2^(*F*_o_^2^) + (0.041*P*)^2^ + 0.8461*P*] where *P* = (*F*_o_^2^ + 2*F*_c_^2^)/3
3678 reflections	(Δ/σ)_max_ < 0.001
251 parameters	Δρ_max_ = 0.36 e Å^−^^3^
19 restraints	Δρ_min_ = −0.33 e Å^−^^3^

### 3-(2-Chloro-6-fluorophenyl)-1,5-bis(thiophen-2-yl)pentane-1,5-dione Special details

**Table:**

Geometry. All esds (except the esd in the dihedral angle between two l.s. planes) are estimated using the full covariance matrix. The cell esds are taken into account individually in the estimation of esds in distances, angles and torsion angles; correlations between esds in cell parameters are only used when they are defined by crystal symmetry. An approximate (isotropic) treatment of cell esds is used for estimating esds involving l.s. planes.

### 3-(2-Chloro-6-fluorophenyl)-1,5-bis(thiophen-2-yl)pentane-1,5-dione Fractional atomic coordinates and isotropic or equivalent isotropic displacement parameters (Å^2^)

**Table:**

	*x*	*y*	*z*	*U*_iso_*/*U*_eq_	Occ. (<1)
S1	0.48547 (5)	1.11343 (9)	0.68846 (5)	0.0347 (2)	
S2	0.17955 (5)	0.15490 (9)	0.57749 (4)	0.02808 (18)	
O1	0.35484 (12)	0.8198 (2)	0.63059 (11)	0.0325 (5)	
O2	0.20623 (11)	0.2763 (2)	0.40727 (11)	0.0257 (4)	
C1	0.23408 (15)	0.7006 (3)	0.45142 (15)	0.0185 (5)	0.931 (4)
H1	0.208985	0.704636	0.500503	0.022*	0.931 (4)
C1A	0.23408 (15)	0.7006 (3)	0.45142 (15)	0.0185 (5)	0.069 (4)
H1A	0.216615	0.704850	0.506277	0.022*	0.069 (4)
C2	0.29032 (16)	0.8652 (3)	0.46245 (16)	0.0211 (5)	
H2A	0.324906	0.852587	0.423181	0.025*	
H2B	0.249983	0.965811	0.437873	0.025*	
C3	0.35410 (16)	0.9041 (3)	0.56334 (16)	0.0222 (6)	
C4	0.41533 (16)	1.0496 (3)	0.57579 (16)	0.0216 (6)	
C5	0.53198 (18)	1.2697 (3)	0.6448 (2)	0.0334 (7)	
H5	0.577736	1.345670	0.683339	0.040*	
C6	0.49693 (17)	1.2742 (3)	0.54950 (19)	0.0296 (6)	
H6	0.515453	1.352724	0.513567	0.036*	
C7	0.42941 (17)	1.1482 (3)	0.50947 (18)	0.0256 (6)	
H7	0.397187	1.133687	0.443422	0.031*	
C8	0.29140 (16)	0.5332 (3)	0.46852 (16)	0.0195 (5)	
H8A	0.310864	0.518754	0.416146	0.023*	
H8B	0.345162	0.546509	0.527707	0.023*	
C9	0.24180 (15)	0.3726 (3)	0.47531 (16)	0.0192 (5)	
C10	0.23621 (16)	0.3363 (3)	0.56578 (16)	0.0206 (5)	
C11	0.20209 (19)	0.2051 (3)	0.69187 (18)	0.0319 (7)	
H11	0.183088	0.136201	0.731254	0.038*	
C12	0.24999 (19)	0.3535 (4)	0.72113 (17)	0.0328 (7)	
H12	0.267969	0.400652	0.782783	0.039*	
C13	0.26995 (18)	0.4295 (3)	0.64853 (17)	0.0300 (6)	
H13	0.303128	0.533825	0.656235	0.036*	
F1	0.2498 (2)	0.7339 (4)	0.2741 (3)	0.0361 (9)	0.931 (4)
Cl1	0.04217 (6)	0.63824 (16)	0.44021 (6)	0.0390 (3)	0.931 (4)
C14	0.15660 (18)	0.6954 (3)	0.35533 (18)	0.0185 (7)	0.931 (4)
C15	0.1675 (2)	0.7152 (5)	0.2710 (2)	0.0244 (8)	0.931 (4)
C16	0.1007 (2)	0.7139 (5)	0.1826 (2)	0.0326 (8)	0.931 (4)
H16	0.112992	0.728642	0.128086	0.039*	0.931 (4)
C17	0.0151 (2)	0.6907 (5)	0.1749 (2)	0.0361 (8)	0.931 (4)
H17	−0.032523	0.690595	0.114242	0.043*	0.931 (4)
C18	−0.0023 (2)	0.6679 (4)	0.2535 (3)	0.0328 (8)	0.931 (4)
H18	−0.061441	0.650549	0.247814	0.039*	0.931 (4)
C19	0.06845 (18)	0.6705 (4)	0.3424 (2)	0.0230 (7)	0.931 (4)
F1A	0.068 (3)	0.647 (5)	0.466 (2)	0.0361 (9)	0.069 (4)
Cl1A	0.2623 (17)	0.701 (4)	0.2830 (19)	0.0390 (3)	0.069 (4)
C14A	0.1464 (16)	0.672 (3)	0.366 (2)	0.0185 (7)	0.069 (4)
C15A	0.155 (2)	0.687 (8)	0.281 (3)	0.0244 (8)	0.069 (4)
C16A	0.078 (3)	0.687 (7)	0.200 (3)	0.0326 (8)	0.069 (4)
H16A	0.080506	0.698995	0.140011	0.039*	0.069 (4)
C17A	−0.002 (3)	0.669 (8)	0.206 (3)	0.0361 (8)	0.069 (4)
H17A	−0.054819	0.663755	0.150898	0.043*	0.069 (4)
C18A	−0.005 (3)	0.657 (6)	0.293 (3)	0.0328 (8)	0.069 (4)
H18A	−0.061667	0.648735	0.294035	0.039*	0.069 (4)
C19A	0.068 (2)	0.658 (6)	0.380 (2)	0.0230 (7)	0.069 (4)

### 3-(2-Chloro-6-fluorophenyl)-1,5-bis(thiophen-2-yl)pentane-1,5-dione Atomic displacement parameters (Å^2^)

**Table:**

	*U*^11^	*U*^22^	*U*^33^	*U*^12^	*U*^13^	*U*^23^
S1	0.0333 (4)	0.0364 (4)	0.0268 (4)	−0.0097 (3)	0.0041 (3)	−0.0050 (3)
S2	0.0367 (4)	0.0245 (4)	0.0261 (3)	−0.0031 (3)	0.0158 (3)	0.0013 (3)
O1	0.0392 (12)	0.0314 (11)	0.0211 (9)	−0.0098 (9)	0.0061 (9)	0.0016 (8)
O2	0.0330 (11)	0.0240 (10)	0.0188 (9)	−0.0017 (8)	0.0091 (8)	−0.0026 (8)
C1	0.0195 (13)	0.0200 (13)	0.0167 (12)	0.0002 (11)	0.0080 (11)	0.0002 (10)
C1A	0.0195 (13)	0.0200 (13)	0.0167 (12)	0.0002 (11)	0.0080 (11)	0.0002 (10)
C2	0.0231 (14)	0.0188 (13)	0.0197 (12)	0.0000 (11)	0.0069 (11)	0.0005 (10)
C3	0.0216 (14)	0.0219 (13)	0.0227 (13)	0.0035 (11)	0.0085 (11)	0.0009 (11)
C4	0.0183 (13)	0.0193 (13)	0.0234 (13)	0.0021 (11)	0.0044 (11)	−0.0029 (11)
C5	0.0271 (16)	0.0265 (15)	0.0428 (17)	−0.0046 (13)	0.0102 (14)	−0.0078 (13)
C6	0.0267 (15)	0.0207 (14)	0.0433 (17)	−0.0006 (12)	0.0160 (14)	−0.0002 (12)
C7	0.0256 (15)	0.0238 (14)	0.0260 (13)	0.0024 (12)	0.0090 (12)	−0.0021 (11)
C8	0.0186 (13)	0.0226 (13)	0.0165 (12)	0.0017 (11)	0.0063 (11)	0.0018 (10)
C9	0.0158 (13)	0.0193 (13)	0.0187 (13)	0.0051 (11)	0.0030 (11)	0.0017 (10)
C10	0.0233 (14)	0.0171 (13)	0.0214 (13)	0.0019 (11)	0.0090 (11)	0.0025 (10)
C11	0.0451 (18)	0.0285 (16)	0.0260 (14)	0.0054 (14)	0.0184 (14)	0.0080 (12)
C12	0.0513 (19)	0.0312 (16)	0.0178 (13)	−0.0009 (14)	0.0157 (13)	0.0027 (11)
C13	0.0378 (17)	0.0260 (15)	0.0212 (13)	−0.0053 (13)	0.0066 (13)	−0.0003 (11)
F1	0.0400 (19)	0.0373 (17)	0.0408 (15)	0.0063 (11)	0.0263 (13)	0.0092 (11)
Cl1	0.0258 (6)	0.0652 (6)	0.0289 (5)	−0.0084 (5)	0.0141 (4)	−0.0055 (5)
C14	0.0194 (14)	0.0133 (13)	0.0198 (14)	0.0019 (11)	0.0049 (12)	0.0009 (10)
C15	0.0316 (17)	0.021 (2)	0.0197 (15)	0.0010 (14)	0.0093 (13)	0.0025 (12)
C16	0.047 (2)	0.0335 (19)	0.0164 (16)	0.0068 (16)	0.0118 (14)	0.0048 (13)
C17	0.035 (2)	0.045 (2)	0.0165 (17)	0.0075 (16)	−0.0020 (15)	0.0014 (15)
C18	0.0251 (16)	0.0401 (18)	0.0277 (19)	0.0020 (14)	0.0048 (17)	−0.0037 (18)
C19	0.0254 (16)	0.0236 (15)	0.0182 (16)	0.0024 (12)	0.0068 (14)	−0.0012 (13)
F1A	0.0400 (19)	0.0373 (17)	0.0408 (15)	0.0063 (11)	0.0263 (13)	0.0092 (11)
Cl1A	0.0258 (6)	0.0652 (6)	0.0289 (5)	−0.0084 (5)	0.0141 (4)	−0.0055 (5)
C14A	0.0194 (14)	0.0133 (13)	0.0198 (14)	0.0019 (11)	0.0049 (12)	0.0009 (10)
C15A	0.0316 (17)	0.021 (2)	0.0197 (15)	0.0010 (14)	0.0093 (13)	0.0025 (12)
C16A	0.047 (2)	0.0335 (19)	0.0164 (16)	0.0068 (16)	0.0118 (14)	0.0048 (13)
C17A	0.035 (2)	0.045 (2)	0.0165 (17)	0.0075 (16)	−0.0020 (15)	0.0014 (15)
C18A	0.0251 (16)	0.0401 (18)	0.0277 (19)	0.0020 (14)	0.0048 (17)	−0.0037 (18)
C19A	0.0254 (16)	0.0236 (15)	0.0182 (16)	0.0024 (12)	0.0068 (14)	−0.0012 (13)

### 3-(2-Chloro-6-fluorophenyl)-1,5-bis(thiophen-2-yl)pentane-1,5-dione Geometric parameters (Å, º)

**Table:**

S1—C5	1.703 (3)	C10—C13	1.375 (3)
S1—C4	1.731 (2)	C11—C12	1.357 (4)
S2—C11	1.700 (3)	C11—H11	0.9500
S2—C10	1.727 (2)	C12—C13	1.417 (3)
O1—C3	1.222 (3)	C12—H12	0.9500
O2—C9	1.226 (3)	C13—H13	0.9500
C1—C14	1.521 (3)	F1—C15	1.342 (5)
C1—C2	1.536 (3)	Cl1—C19	1.750 (3)
C1—C8	1.554 (3)	C14—C19	1.395 (4)
C1—H1	1.0000	C14—C15	1.395 (3)
C1A—C14A	1.529 (19)	C15—C16	1.368 (4)
C1A—C2	1.536 (3)	C16—C17	1.376 (5)
C1A—C8	1.554 (3)	C16—H16	0.9500
C1A—H1A	1.0000	C17—C18	1.368 (4)
C2—C3	1.520 (3)	C17—H17	0.9500
C2—H2A	0.9900	C18—C19	1.401 (4)
C2—H2B	0.9900	C18—H18	0.9500
C3—C4	1.465 (3)	F1A—C19A	1.341 (19)
C4—C7	1.369 (3)	Cl1A—C15A	1.75 (2)
C5—C6	1.350 (4)	C14A—C15A	1.390 (19)
C5—H5	0.9500	C14A—C19A	1.39 (2)
C6—C7	1.415 (4)	C15A—C16A	1.37 (2)
C6—H6	0.9500	C16A—C17A	1.36 (2)
C7—H7	0.9500	C16A—H16A	0.9500
C8—C9	1.507 (3)	C17A—C18A	1.36 (2)
C8—H8A	0.9900	C17A—H17A	0.9500
C8—H8B	0.9900	C18A—C19A	1.39 (2)
C9—C10	1.466 (3)	C18A—H18A	0.9500
			
C5—S1—C4	91.38 (13)	C13—C10—S2	110.85 (18)
C11—S2—C10	91.43 (12)	C9—C10—S2	119.83 (17)
C14—C1—C2	111.15 (19)	C12—C11—S2	113.1 (2)
C14—C1—C8	110.94 (19)	C12—C11—H11	123.4
C2—C1—C8	111.47 (19)	S2—C11—H11	123.4
C14—C1—H1	107.7	C11—C12—C13	111.8 (2)
C2—C1—H1	107.7	C11—C12—H12	124.1
C8—C1—H1	107.7	C13—C12—H12	124.1
C14A—C1A—C2	122.0 (10)	C10—C13—C12	112.8 (2)
C14A—C1A—C8	109.1 (9)	C10—C13—H13	123.6
C2—C1A—C8	111.47 (19)	C12—C13—H13	123.6
C14A—C1A—H1A	104.1	C19—C14—C15	113.4 (2)
C2—C1A—H1A	104.1	C19—C14—C1	123.9 (2)
C8—C1A—H1A	104.1	C15—C14—C1	122.7 (3)
C3—C2—C1	114.36 (19)	F1—C15—C16	115.5 (3)
C3—C2—C1A	114.36 (19)	F1—C15—C14	119.0 (3)
C3—C2—H2A	108.7	C16—C15—C14	125.5 (3)
C1—C2—H2A	108.7	C15—C16—C17	118.1 (3)
C3—C2—H2B	108.7	C15—C16—H16	121.0
C1—C2—H2B	108.7	C17—C16—H16	121.0
H2A—C2—H2B	107.6	C18—C17—C16	120.8 (3)
O1—C3—C4	121.5 (2)	C18—C17—H17	119.6
O1—C3—C2	122.5 (2)	C16—C17—H17	119.6
C4—C3—C2	116.1 (2)	C17—C18—C19	118.9 (3)
C7—C4—C3	129.7 (2)	C17—C18—H18	120.5
C7—C4—S1	110.67 (19)	C19—C18—H18	120.5
C3—C4—S1	119.58 (18)	C14—C19—C18	123.2 (3)
C6—C5—S1	112.9 (2)	C14—C19—Cl1	120.0 (2)
C6—C5—H5	123.6	C18—C19—Cl1	116.8 (2)
S1—C5—H5	123.6	C15A—C14A—C19A	127 (2)
C5—C6—C7	112.1 (2)	C15A—C14A—C1A	113 (3)
C5—C6—H6	124.0	C19A—C14A—C1A	119 (3)
C7—C6—H6	124.0	C16A—C15A—C14A	117 (3)
C4—C7—C6	113.0 (2)	C16A—C15A—Cl1A	125 (3)
C4—C7—H7	123.5	C14A—C15A—Cl1A	118 (3)
C6—C7—H7	123.5	C17A—C16A—C15A	120 (4)
C9—C8—C1	112.44 (19)	C17A—C16A—H16A	120.1
C9—C8—C1A	112.44 (19)	C15A—C16A—H16A	120.1
C9—C8—H8A	109.1	C18A—C17A—C16A	120 (5)
C1—C8—H8A	109.1	C18A—C17A—H17A	120.2
C9—C8—H8B	109.1	C16A—C17A—H17A	120.2
C1—C8—H8B	109.1	C17A—C18A—C19A	126 (5)
H8A—C8—H8B	107.8	C17A—C18A—H18A	116.8
O2—C9—C10	120.9 (2)	C19A—C18A—H18A	116.8
O2—C9—C8	121.3 (2)	F1A—C19A—C14A	122 (3)
C10—C9—C8	117.8 (2)	F1A—C19A—C18A	128 (4)
C13—C10—C9	129.3 (2)	C14A—C19A—C18A	110 (3)
			
C14—C1—C2—C3	165.2 (2)	C11—C12—C13—C10	0.2 (4)
C8—C1—C2—C3	−70.4 (2)	C2—C1—C14—C19	−127.6 (3)
C14A—C1A—C2—C3	158.3 (16)	C8—C1—C14—C19	107.8 (3)
C8—C1A—C2—C3	−70.4 (2)	C2—C1—C14—C15	52.4 (3)
C1—C2—C3—O1	−6.0 (3)	C8—C1—C14—C15	−72.2 (3)
C1A—C2—C3—O1	−6.0 (3)	C19—C14—C15—F1	−177.7 (3)
C1—C2—C3—C4	174.0 (2)	C1—C14—C15—F1	2.2 (5)
C1A—C2—C3—C4	174.0 (2)	C19—C14—C15—C16	0.9 (5)
O1—C3—C4—C7	173.8 (3)	C1—C14—C15—C16	−179.2 (3)
C2—C3—C4—C7	−6.1 (4)	F1—C15—C16—C17	178.4 (3)
O1—C3—C4—S1	−4.3 (3)	C14—C15—C16—C17	−0.2 (6)
C2—C3—C4—S1	175.73 (17)	C15—C16—C17—C18	−0.6 (5)
C5—S1—C4—C7	−0.1 (2)	C16—C17—C18—C19	0.8 (5)
C5—S1—C4—C3	178.4 (2)	C15—C14—C19—C18	−0.7 (4)
C4—S1—C5—C6	−0.3 (2)	C1—C14—C19—C18	179.3 (3)
S1—C5—C6—C7	0.5 (3)	C15—C14—C19—Cl1	178.5 (2)
C3—C4—C7—C6	−177.9 (2)	C1—C14—C19—Cl1	−1.4 (4)
S1—C4—C7—C6	0.4 (3)	C17—C18—C19—C14	0.0 (5)
C5—C6—C7—C4	−0.6 (3)	C17—C18—C19—Cl1	−179.3 (2)
C14—C1—C8—C9	−65.8 (3)	C2—C1A—C14A—C15A	55 (3)
C2—C1—C8—C9	169.78 (19)	C8—C1A—C14A—C15A	−78 (3)
C14A—C1A—C8—C9	−52.7 (15)	C2—C1A—C14A—C19A	−118 (2)
C2—C1A—C8—C9	169.78 (19)	C8—C1A—C14A—C19A	110 (2)
C1—C8—C9—O2	97.9 (3)	C19A—C14A—C15A—C16A	0 (7)
C1A—C8—C9—O2	97.9 (3)	C1A—C14A—C15A—C16A	−172 (4)
C1—C8—C9—C10	−81.2 (2)	C19A—C14A—C15A—Cl1A	−179 (3)
C1A—C8—C9—C10	−81.2 (2)	C1A—C14A—C15A—Cl1A	9 (5)
O2—C9—C10—C13	−178.8 (3)	C14A—C15A—C16A—C17A	−2 (8)
C8—C9—C10—C13	0.4 (4)	Cl1A—C15A—C16A—C17A	178 (4)
O2—C9—C10—S2	0.5 (3)	C15A—C16A—C17A—C18A	2 (8)
C8—C9—C10—S2	179.57 (17)	C16A—C17A—C18A—C19A	−2 (9)
C11—S2—C10—C13	−0.3 (2)	C15A—C14A—C19A—F1A	−179 (4)
C11—S2—C10—C9	−179.7 (2)	C1A—C14A—C19A—F1A	−8 (5)
C10—S2—C11—C12	0.4 (2)	C15A—C14A—C19A—C18A	0 (6)
S2—C11—C12—C13	−0.4 (3)	C1A—C14A—C19A—C18A	172 (3)
C9—C10—C13—C12	179.4 (2)	C17A—C18A—C19A—F1A	180 (5)
S2—C10—C13—C12	0.1 (3)	C17A—C18A—C19A—C14A	0 (7)

### 3-(2-Chloro-6-fluorophenyl)-1,5-bis(thiophen-2-yl)pentane-1,5-dione Hydrogen-bond geometry (Å, º)

**Table:**

*D*—H···*A*	*D*—H	H···*A*	*D*···*A*	*D*—H···*A*
C1—H1···Cl1	1.00	2.57	3.128 (3)	115
C1—H1···F1*A*	1.00	2.21	2.86 (5)	122
C2—H2*A*···Cl1*A*	0.99	2.31	2.91 (3)	118
C2—H2*A*···F1	0.99	2.32	2.898 (5)	116
C2—H2*B*···O2^i^	0.99	2.48	3.417 (3)	157
C5—H5···Cl1*A*^ii^	0.95	2.49	3.12 (3)	123
C8—H8*A*···Cl1*A*	0.99	2.35	3.01 (3)	123
C13—H13···O1	0.95	2.45	3.367 (3)	163
C16—H16···F1*A*^iii^	0.95	2.50	3.35 (3)	149

Symmetry codes: (i) *x*, *y*+1, *z*; (ii) −*x*+1, −*y*+2, −*z*+1; (iii) *x*, −*y*+3/2, *z*−1/2.
