# Supplementary material for: Crystal structure and Hirshfeld surface analysis of 3-(2-chloro-6-fluoro­phen­yl)-1,5-bis­(thio­phen-2-yl)pentane-1,5-dione
Source: Acta Crystallogr E Crystallogr Commun. 2025 Oct 31;81(Pt 11):1094–8. doi: 10.1107/S2056989025009284 (PMC12589841; doi:10.1107/S2056989025009284)
Supplement: Supplementary file 3 [file e-81-01094-sup3.pdf]

The Supporting Information file of oi2026

Crystal structures and Hirshfeld surface analyses of 3-(2-chloro-6-fluorophenyl)-1,5-di(thiophen-2-yl)pentane-1,5-dione

Atash V. Gurbanov,[a] Mehmet Akkurt,[b] Nurlana D. Sadikhova[c] and Gizachew Mulugeta Manahelohe[d]*

[a]Excellence Center, Baku State University, Z. Khalilov Str. 33, AZ 1148, Baku, Azerbaijan, [b]Department of Physics, Faculty of Sciences, Erciyes University, 38039 Kayseri, Turkey, [c]Department of Organic Chemistry, Baku State University, Z. Khalilov Str. 33, AZ 1148, Baku, Azerbaijan, and [d]Department of Chemistry, University of Gondar, P.O. Box 196, Gondar, Ethiopia

Correspondence e-mail: Gizachew.Mulugeta@uog.edu.et

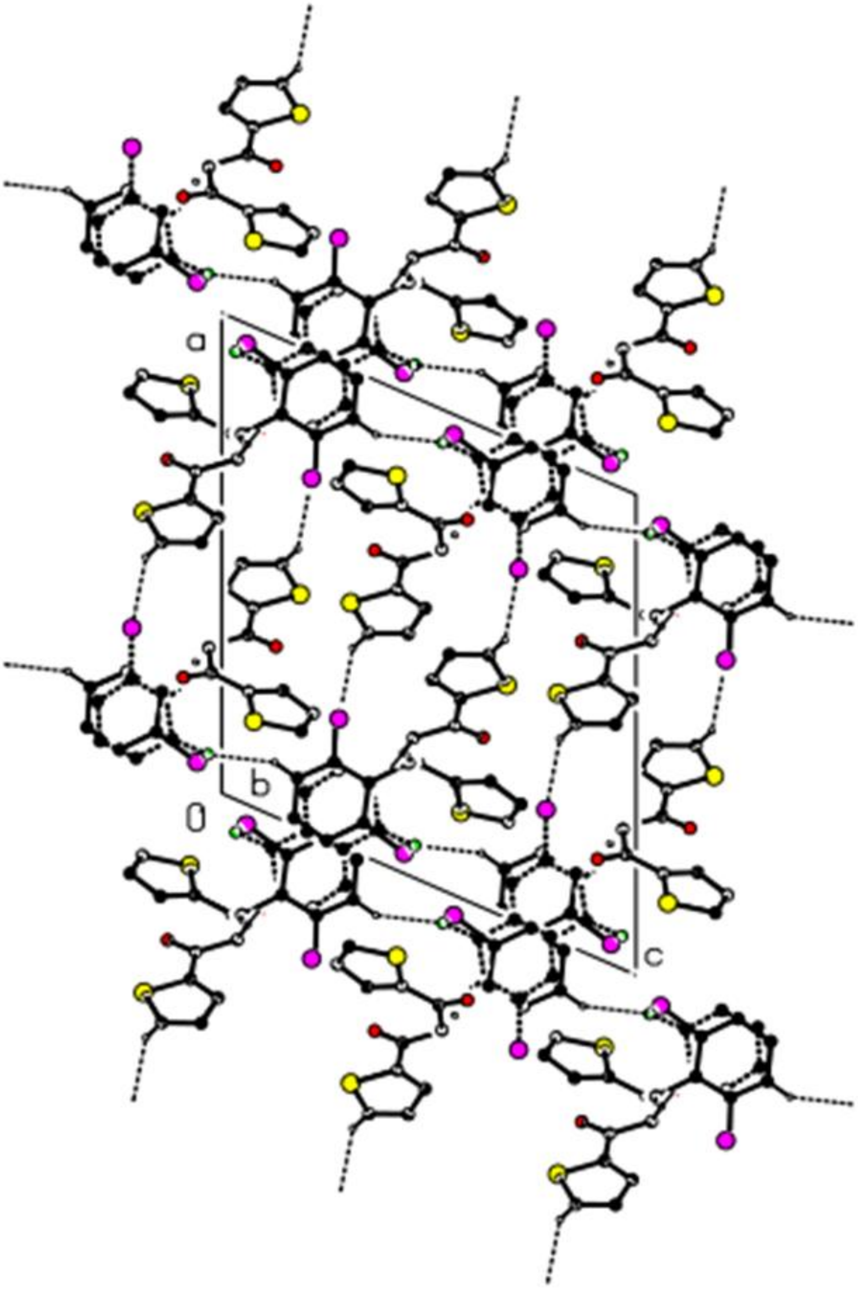

Sup-Fig. 1. View of molecular packing down the [010] direction, revealing main and minor disorder components and two-dimensional network-forming C—H⋯O, C—H⋯Cl, and C—H⋯F intermolecular interactions.

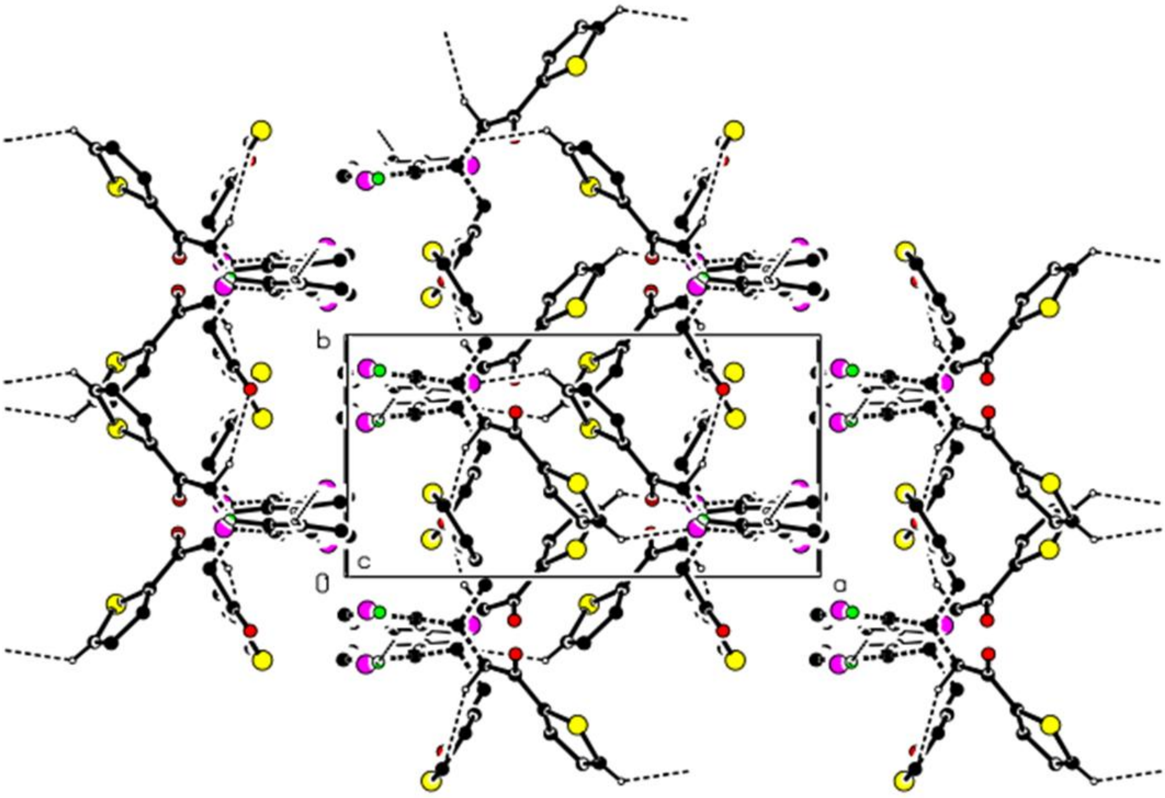

Sup-Fig. 2. View of the molecular packing in Sup-Fig. 1 along the [001] direction.
